# Single Transparent Piezoelectric Detector for Optoacoustic Sensing—Design and Signal Processing

**DOI:** 10.3390/s19092195

**Published:** 2019-05-12

**Authors:** Elias Blumenröther, Oliver Melchert, Jonas Kanngießer, Merve Wollweber, Bernhard Roth

**Affiliations:** 1 Hannover Centre for Optical Technologies, Nienburger Straße 17, 30167 Hannover, Germany; elias.blumenroether@hot.uni-hannover.de (E.B.); jonas.kanngiesser@hot.uni-hannover.de (J.K.); M.Wollweber@lzh.de (M.W.); 2 Cluster of Excellence PhoenixD, Leibniz University Hannover, Welfengarten 1, 30167 Hannover, Germany; oliver.melchert@hot.uni-hannover.de; 3 Institut für Quantenoptik, Leibniz University Hannover, Welfengarten 1, 30167 Hannover, Germany; 4 Laser Zentrum Hannover e.V., Industrial and Biomedical Optics Department, Hollerithallee 8, 30419 Hannover, Germany

**Keywords:** optoacoustics, acoustic near field, handheld, transparent detector

## Abstract

In this article, we present a simple and intuitive approach to create a handheld optoacoustic setup for near field measurements. A single piezoelectric transducer glued in between two sheets of polymethyl methacrylate (PMMA) facilitates nearfield depth profiling of layered media. The detector electrodes are made of indium tin oxide (ITO) which is both electrically conducting as well as optically transparent, enabling an on-axis illumination through the detector. By mapping the active detector area, we show that it matches the design form precisely. We also present a straightforward approach to determine the instrument response function, which allows to obtain the original pressure profile arriving at the detector. To demonstrate the validity of this approach, the measurement on a simple test sample is deconvolved with the instrument response function and compared to simulation results. Except for the sputter instrumentation, all required materials and instruments as well as the tools needed to create such a setup are available to standard scientific laboratories.

## 1. Introduction

In the past decade, great progress was made in the field of optoacoustics (OAs). Elaborate setups for OA microscopy produce high resolution images of biological tissue in depths beyond the optical diffusion length [1,2,3,4]. As regular ultrasound transducers use metal electrodes, the light and sound paths must be separated to prevent shadowing in either one of them. To avoid these difficulties, transparent transducers have been created by using saline solution as electrodes [5] or even by using a micro ring resonator to record the changes in pressure [6]. Another approach is to detect the deformation caused by stress waves via a Fabry-Pérot interferometer [7] which facilitates a setup that works purely optically in this case. While these approaches are promising, novel sensor solutions that offer high sensitivity and robustness, allow easy and cheap fabrication, are flexible in form and design, or provide extra functionality are still attractive goals for further sensor development. Regular polyvinylidene fluoride (PVDF) transducers are well understood and offer a high sensitivity without the need of complicated setups. Niederhauser et al. have shown the feasibility of a transparent piezoelectric OA detector which consists of two indium tin oxide (ITO) electrodes sputtered on both sides of a PVDF film [8]. In their work, a stationary setup is used to measure the absorption profile of a liquid dye layer. Nowadays ITO is widely used in organic LEDs (OLEDs), solar panels, liquid crystal displays (LCDs), and touchscreens, which makes it more easily available with continuous progress in both its optical transparency and electrical conductance. Through this transparent transducer, a sample can be illuminated almost homogeneously and without significant shadowing along the optical axis. With this on-axis approach, the detector can be brought in very close vicinity to the sample facilitating near field measurements retaining the original pressure profile without alterations caused by acoustic diffraction [9,10].

In this work, we present a transparent handheld optoacoustic system for near field measurements and demonstrate its capability for OA depth profiling. Transmission measurements confirm the transparency of the detector. The precise extent of the active area is mapped by scanning the pyroelectric signal which is created by absorption in the detector itself. Furthermore, we show an intuitive approach to directly obtain the instrument response function which is needed to remove alterations induced by the setup on the measured OA signals. All the materials and tools needed for the handheld system and the validation measurements, with the exception of the sputtering system required to create the electrodes are available to standard scientific laboratories. With regard to the electrode material used, it is known that the pyroelectric background signal of ITO is five times lower than that of aluminum electrodes [8]. Still, the thermal background signal is in the order of magnitude of the OA signals. In our work, we show that this pyroelectric effect can be used to map the active area of the piezoelectric sensor to validate the actual size of the active OA detector area. Also, the system developed is suited to investigate samples of various size and geometry, as is the case, e.g., for in vivo measurement of human skin lesions, which we aim at in the future [11]. In addition, the transparent detector allows manual positioning by eye which is also required by the envisioned use.

This article is structured as follows: First the general setup is explained. This is followed by an in-depth description of the OA detector film as well as its design and fabrication. Then, we describe the signal processing employed and finally, we present an exemplary measurement on a simple phantom to showcase the feasibility of our approach.

## 2. Experimental Setup

### 2.1. Handheld Optoacoustic Setup

**Figure 1:**
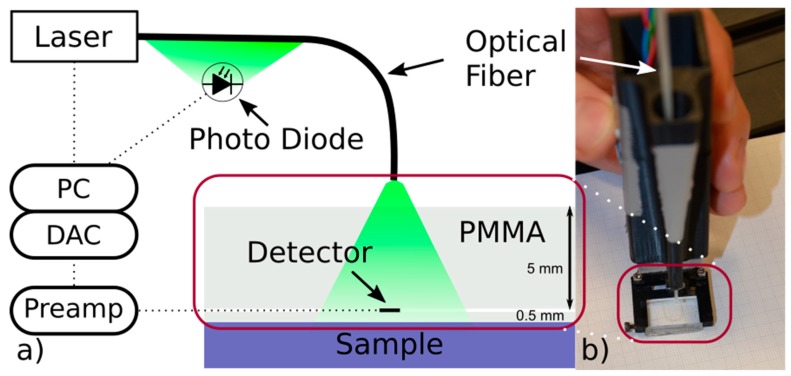
(**a**) Sketch of the optoacoustics (OA) setup. DAC: Data acquisition card, Preamp: Preamplifier. (**b**) Photograph of the probe. The laser injects 532 nm pulses into a fiber with a transparent jacket. A calibrated diode placed next to the fiber monitors the energy. The transparent detector is placed directly on the sample. Illumination is centered through the detector. The polymethyl methacrylate (PMMA) backing layer is 5 mm and the fronting layer 0.5 mm thick.

The OA setup is fiber based to ensure the necessary flexibility for positioning and illumination, see Figure 1. The transparent PVDF-based OA detector is connected to the self-built electrical instrument amplifier with constant amplification of 6 dB in the frequency range from DC to 200 MHz. The data acquisition card (Agilent U1065A, Santa Rosa, CA, USA) has a sample rate of up to 8 Giga samples per second and is capable to resolve the frequencies present in the signal. To monitor the energy of the laser pulses during measurements, a photo diode is placed close to the transparent jacket of the fiber. The photo diode measurements are calibrated to a pyroelectric energy detector positioned in front of the fiber facet in place of the sample.

In earlier stages of the experimental setup, we used poly(vinyl alcohol) hydrogel as backing layer [12] which possesses acoustic properties very similar to living tissue. However, the handling of the hydrogel is impractical when aiming for a handheld device. Instead, the PVDF detector film is glued in between two sheets of PMMA by using optical adhesive (NOA85V, Norland Products, Cranbury, NJ, USA). The “backing” layer reduces the acoustic reflections inside the PVDF film and is thick enough (5 mm) to prevent the reflections at the PMMA-air boundary to interfere with the signal of the region of interest. The “fronting” layer (0.5 mm), named analogously to the “backing” layer, is most important for the reproducibility and reliability, because it ensures a fixed distance between detector and sample in addition to serving as a robust protection layer for the thin detector film. Using the PMMA block, the detector can be fixed to the handheld setup which would be difficult with the flexible detector film alone. The whole frame of the handheld setup is 3D-printed (see Figure 1, right) using Acrylonitrile Butadiene Styrene (Replicator 2X, MakerBot Industries, Brooklyn, NY, USA). For increased visibility, the frame holding the detector is open to one side.

As will be described later in this section, the active area is transparent but with finite absorption. That way, it is possible to see the position of the detector area by eye. Note that due to the high transparency, it takes relatively bright light and the right viewing angle to determine the exact position.

Above the detector, the illumination fiber (WF 600/660N, CeramOptec, Bonn, Germany) is placed inside a drilling hole that is tight enough to hold the fiber in place throughout the measurement without need for further fixation. For the experiment presented here, the fiber facet was positioned 18 mm above the sample, resulting in a beam diameter of approximately 6 mm at the sample surface, which is large enough to ensure near field conditions. A frequency doubled Nd:YAG laser (Ultra 50, Quantel laser, Köln, Germany) provides 8 ns pulses at 532 nm wavelength. The energy density at the surface is approximately 50 $\frac{J}{m^{2}}$. Distilled water is used to ensure acoustic coupling between fronting layer and sample. Ultrasound gel could be used as well. However, it is easier to obtain a thin coupling layer without air bubbles using low viscosity fluids such as water.

### 2.2. Design and Fabrication of the Optoacoustic Detector Film

**Figure 2:**
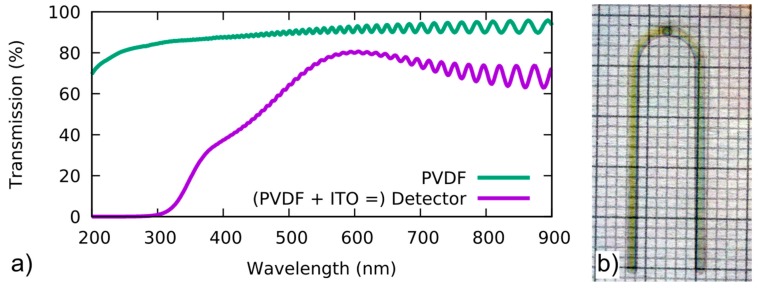
(**a**) Transmission spectra of the polyvinylidene (PVDF) film with and without indium tin oxide (ITO) electrodes. ITO reduces transparency drastically below 400 nm, at 600 nm transmission is highest (80%) and decreases slowly towards higher wavelengths. At 532 nm transmission is still above 70%. Oscillations are due to interferometric effects of the thin film. (**b**) Photo of detector film on scale paper with 1 mm between lines. Contrast is enhanced for visibility. The active area of the detector, where the two electrodes overlap, shows as a slightly darker spot at the apex of the curved electrode section.

**Figure 3:**
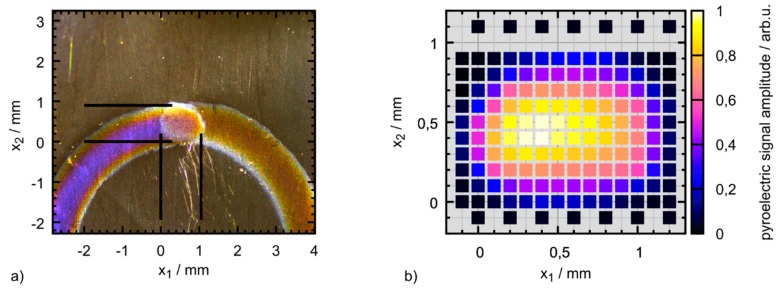
(**a**) Microscopic view of the sputtered indium tin oxide (ITO) electrodes. The electrode sputtered on top of the polyvinylidene (PVDF) film (left) and the electrode at the bottom (right) overlap in a nearly circular region with a diameter of roughly 1 mm. This is expected to be the active area of the piezoelectric sensor. (**b**) Spatial pyroelectric response of the PVDF-sensor. The sensor film has been illuminated through a 150 µm aperture. The absorption within the detector material gives rise to a transient pyroelectric signal. By scanning the illuminated area in x_1_ and x_2_ direction over the sensor film, the energy-normalized amplitude of the pyroelectric signal at different positions of the detector was acquired. The shape and size of the acquired spatial response nicely resembles the area where the sputtered electrodes overlap. Outside of the displayed region no significant pyroelectric signal could be observed.

The centerpiece of the detector setup is a transparent 9 µm thick biaxially poled polyvinylidene fluoride (PVDF) film (Precision Acoustics, Dorchester, UK). On both sides of the piezoelectric film, a thin layer of indium tin oxide (ITO) was sputtered. The sputtering was performed at the Institut für Hochfrequenztechnik (IHF) in Braunschweig. The form of the electrodes was cut into a sheet of metal to produce the sputter mask. By design, the electrodes form a circular region of overlap which constitutes the active area of the detector, see Figure 2b. A microscopic view of the active sensor area can be seen in Figure 3a (image taken with a calibrated Discovery V8 microscope, Zeiss, Germany) To prevent voltage spikes and reflections inside the electrodes, the shape was chosen to be smoothly curved without sharp corners. For a more compact design, the conductive paths could be brought closer together, however, this would increase the crosstalk and the parasitic capacity. Spectrophotometer (Uvikon 931, Kontron Instruments, Rossdorf, Germany) measurements, see Figure 2, show that the transmission through the film is well above 60% for most parts of the visible wavelength range both for the sole PVDF film and in combination with the ITO electrodes. The periodic oscillations in the measurement data are caused by interferometric effects of the thin film. Without the ITO electrodes, the transmission spectrum decreases slowly towards shorter wavelengths. The ITO layers on both sides change the spectrum so that transmission becomes negligible below 300 nm. With increasing wavelength, the detector becomes more transparent with a maximal value of 80% at 600 nm. For even higher wavelengths, the transmission decreases slowly. The only source to produce pulses of sufficient energy at 600 nm are optical parametric oscillators (OPOs) which in many cases are cumbersome and, thus, inconvenient for portable devices. For that reason, we opted for a frequency doubled Nd:YAG at 532 nm. Lasers emitting at wavelengths above 600 nm are available as well, however, besides the transmission through the detector, the absorptive contrast of the sample must be considered. Most samples, and biological tissue in particular, have higher absorption at lower wavelengths, producing stronger OA signals.

### 2.3. Mapping the Active Area of the Detector

**Figure 4:**
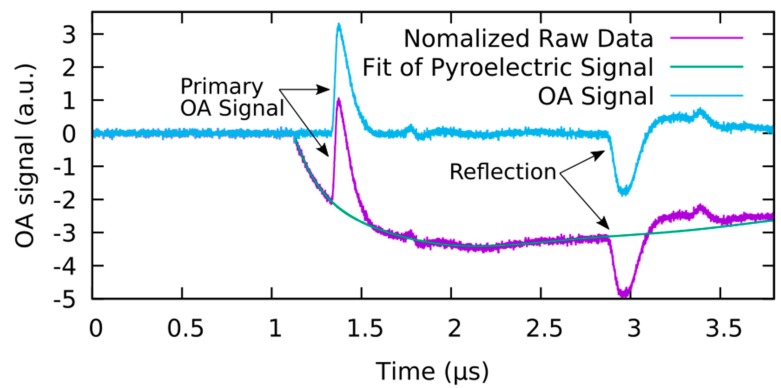
Removal of the pyroelectric signal from the measurement of the instrument response function (IRF). Pyroelectrical signal begins at the time of the laser excitation at 1.12 µs. Primary OA signal arrives at 1.33 µs.

To confirm that the active area of the sensor indeed corresponds to the designed geometry, we make use of the pyroelectric effect that is present in any piezoelectric material. Two conditions must be met to measure a pyroelectric signal. First, the absorption must be finite, which holds true for the whole PVDF film, even without the electrodes (see Figure 2). Second, the pyroelectric effect must induce a voltage between the electrodes to be picked up by the data acquisition card. Thus, only the area where two conductive electrodes overlap produces a measurable pyroelectric signal. The very same argument applies to the piezoelectric detection of a pressure signal with our sensor film. Hence, we consider the detector’s piezoelectric and pyroelectric active area to be equal. We investigated the sensor’s pyroelectric properties using a 150 µm pinhole through which the sensor film was illuminated with short laser pulses (8 ns, λ = 532 nm). A manual micrometer stage (TSMW13-XYZ-1A, Zolix, Beijing, China) allows to laterally scan the pyroelectric response over the detector film. The resulting map is depicted in Figure 3b. Every pixel corresponds to the average of 10 measurements at that position. The color scale is normalized to the maximal amplitude of the pyroelectric signal. An example of a pyroelectric signal can be seen in Figure 4 (green line), where it overlays the OA signal.

Even though there is considerable absorption of the unsputtered PVDF film at the excitation wavelength (see Figure 2), a pyroelectric signal could only be observed when the sensor film was illuminated at the supposed active area, that is, where the electrodes overlap. Furthermore, the active area extends over the whole region of overlap, which suggests that there are no adverse fringe effects reducing the conduction of the ITO layers in close vicinity of the sputter mask/electrode edges. Thereby, we successfully showed that a functioning transparent OA detector of the desired dimensions can be produced reliably. The smooth transition to zero signal amplitude at the edge of the active area in Figure 3b) can be attributed to the illumination spot size of the laser beam of at least 150 µm. The varying distribution in the center is likely caused by inhomogeneities in the ITO thickness. While they do not seem to affect the functionality of the detector, future detector production will aim at a more constant thickness of the ITO layers throughout the active area.

## 3. Measurement of the Instrument Response Function

### 3.1. Creating a Delta Peak Signal

Point spread functions (PSF) or more generally instrument response functions (IRF) represent the effect the measurement setup induces on the signal. The most straightforward way to obtain the IRF is to measure on a sample which produces a delta function as signal. In the case of our setup, a 1D delta function is needed, or in terms of the optoacoustic source, an infinitesimally thin absorbing layer. Thin layers can be created by coating a glass plate with coal or any other strong absorber. Unfortunately, it is difficult to obtain a thin layer of sufficiently high quality which can sustain both the distilled water as well as the mechanical strain during measurements. It is, however, much simpler to use a thick sample with such high absorption that the penetration depth and thus the contributing absorbing layer becomes vanishingly thin. Here, a 1.65 mm thick piece of black plastic was used. Note that, it is important to use a sample which is thick enough that the acoustic reflections occur outside of the relevant measurement time window. Also, the sample should be acoustically homogeneous to prevent reflections inside the sample.

In general, the resulting pressure wave is not a delta function in its purest form. Nonetheless, it can be assumed as such if the signal duration is shorter than the theoretical limit of the resolution. From the physical point of view, the axial resolution is limited by the thickness of the detector. If the spatial extent of the transient signal is smaller than the detector film thickness, it cannot be resolved. For the detector presented here, the thickness is close to 10 µm corresponding to an absorption coefficient of ~$100\;{\mathrm{mm}}^{-1}$ for the smallest resolvable signal length (assuming similar sound velocities of detector and absorber) which is a reasonable value for black plastic (The precise absorption coefficient is too high to be measured). As will be described later, during the data processing of the pure OA signal, frequencies above an acoustic frequency of 20 MHz are suppressed, thus, further reducing the achievable resolution. Using the 20 MHz cutoff frequency and the speed of sound in the plastic, the minimum absorption coefficient required can be roughly approximated as follows: 20 MHz signal frequency corresponds to 50 ns signal duration. During that time, the pressure transient travels 0.11 mm at a sound velocity of 2150 m/s (black plastic). In other words, signals created in a depth of less than 100 µm cannot be resolved. This means that due to the 20 MHz cutoff the minimal absorption coefficient needed is reduced to ~$10\;{\mathrm{mm}}^{-1}$ which is far below the absorption coefficient of the black plastic. Thus, it is justified to use the measurement on the black plastic to deduce the instrument response function.

### 3.2. Removing the Pyroelectric Signal from the Instrument Response Function (IRF)

In the following, the measurement data and the signal processing to obtain the IRF will be presented. Note that the IRF itself, that is, the OA signal of the black plastic, is not the signal of interest but is needed for the deconvolution process to reconstruct the original pressure profile from the raw signal of the sample of interest. Nonetheless, this raw signal can be used to illustrate basic features present in any measurement obtained by this setup. As represented in Figure 4, the raw data shows a broad valley starting ~1.1 µs after the trigger delay. This feature is caused by the pyroelectric effect from the absorption of laser radiation in the detector itself.

The amplitude of the pyroelectric signal is proportional to the energy of the laser; thus, it could be used for energy calibration. Unfortunately, the amplitude and form of the pyroelectric signal also depend on the capacity of the sample, which is hard to control especially when measuring on living samples. For that reason, the precise details of the pyroelectric signal are assumed to be unknown and not part of the measured information. To remove the pyroelectric signal, a polynomial function of 10th order is fitted to the signal and subsequently subtracted. For the fitting process, the region of the actual OA signals was not taken into account. To identify the relevant OA signal, the fronting layer is quite helpful. In the time interval required by the pressure wave to propagate through the fronting layer, only the pyroelectric signal contributes to the raw signal and can easily be fitted. Determining the region between the initial OA signal and its reflections from various boundaries is more complex. However, by comparing the curve to a measurement without a sample, the general progression of the pyroelectric signal can be determined and distinguished from regions where the OA signal or its reflections are present.

Regarding the resulting data curve after the pyroelectric signal removal, all remaining signals can be ascribed to OA pressure waves. Ideally, the initial pulse corresponding to the absorption at the black plastic sample (upper plastic boundary) arrives at the detector after 1.33 µs which is the sum of the laser pulse to DAQ trigger delay (1.12 µs) and the acoustic time-of-flight through the fronting layer (0.21 µs). At approximately 1.8 µs, a minor signal feature is visible, caused by the reflections first off the detector film and then the PMMA-sample boundary. The delay to the original signal corresponds to twice the acoustic time-of-flight within the fronting layer. Finally, slightly before 3 µs, the acoustic wave reflected from the bottom of the black plastic sample arrives, which is followed by the double reflection from the inner boundaries of the fronting layer.

**Figure 5:**
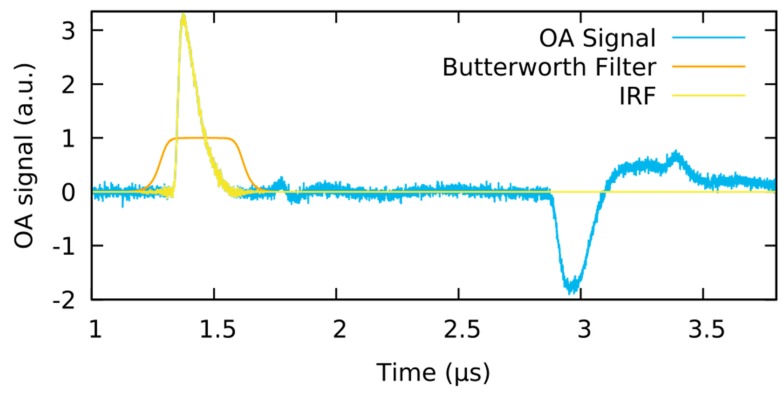
The optoacoustics (OA) signal of black plastic (blue) shows unwanted reflections. Butterworth filter as used on OA signal (orange). Resulting instrument response functions (IRF), only featuring the delta peak response (yellow).

The IRF represents the response of the measurement system to a delta peak signal. Thus, the reflections produced by the boundaries of the black plastic sample must be removed. Therefore, after the pyroelectric signal is removed, the signal data is zero-padded. Anything but the OA signal which corresponds to the delta-peak signal is suppressed. To ensure that no sharp signal cut-offs are created, a Butterworth filter is used (see Figure 5).

Note that the OA transient extends over more than 0.2 µs which would correspond to a depth of about 0.4 mm and is far too broad to represent the absorption profile in the black plastic. While this work focuses on obtaining and processing of the IRF instead of investigating the reasons behind its broadened appearance, it is reasonable that the detector setup acts as a low pass filter. Possible contributors to the signal deformation and broadening are the finite extent of the detector film as well as the electronics of the amplifier. Although the frequency response of the preamplifier has been verified to be constant up to 200 MHz, it is plausible that the high resistance of the ITO electrodes as well as the interplay with the piezoelectric PVDF film suppress high frequencies.

## 4. Results of Raw Signal Processing and Deconvolution

**Figure 6:**
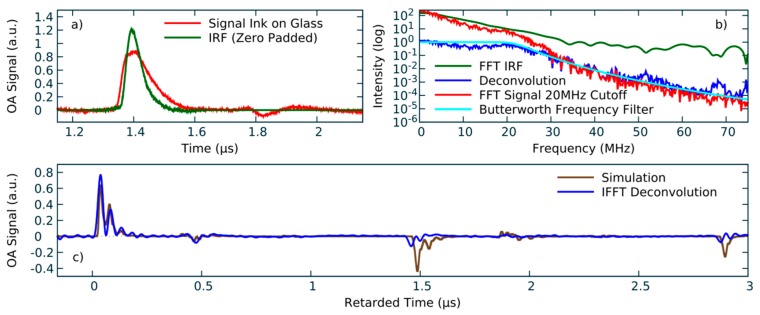
Visualization of post-processing. (**a**) optoacoustic (OA) signal of the instrument response functions (IRF) and ink-on-glass after removal of the pyroelectric signal (green and red). IRF was zero padded to prevent transformation artifacts. (**b**) Frequency spectra of IRF (green) and the ink-on glass-signal with 20 MHz cut-off (red), the Butterworth filter for the 20 MHz cut-off as applied on the signal (turquoise), and the resulting signal after deconvolution (blue). (**c**) Back transformed signal (blue) and 1D simulation (brown) agree well. All features of the signal can be identified.

To showcase that the resulting IRF can be used to reproduce the original pressure signal by deconvolution, we created a simple test sample. Using a permanent board marker, we applied black ink on a 4 mm thick plate of borosilicate glass. Although the thickness of the ink layer cannot be determined by any means available to us, it could be seen by comparison with electrical tape that it is distinctly thinner than 100 µm. To ensure acoustic coupling, a drop of distilled water was applied between the fronting layer of the detector setup and the ink. As explained earlier, after measurement of the raw OA signal, the pyroelectric signal contribution was removed from the signal, both for the IRF measurement as well as from the signal of the ink-on-glass sample. Regarding the IRF, only the main OA signal is relevant, thus the measurement is zero-padded to remove the reflections. To prevent sharp edges that would yield artifacts in the deconvolution, again a time domain Butterworth filter is applied. In Figure 6a, it can be seen that the overall form of the IRF and the signal of the ink are very similar in shape, which is to be expected because both result from measurements on black layers. The main difference lies in the change of acoustic impedance from ink to glass which is absent in the black piece of plastic used for the IRF. By means of a fast Fourier transformation (FFT), both signals are converted to the frequency domain for the deconvolution. Most of the frequency spectrum has to be suppressed in order to prevent noise and parasitic signals to dominate the back-transformed signal. As shown in Figure 6b, a second Butterworth filter is used in the frequency domain to suppress any contributions to the signal with frequencies above 20 MHz. If the cut-off frequency is only a few MHz higher, the resulting signal is dominated by noise. Subsequently the FFT of the ink signal is divided by the FFT of the IRF and back-transformed to obtain the processed pressure profile.

To visualize the validity of the approach, the deconvolved signal is compared with a 1D finite-difference simulation of the ink-on-glass setup. The program code and its description are made available on GitHub [13]. Because acoustic diffraction is a product of propagation in multiple spatial dimensions, it is not present in 1D simulations. The setup is designed to work in the nearfield, so diffraction is negligible. Diffraction only plays a significant role at long runtimes of the signal. It probably contributes to the differing appearances of signal features at times >1.4 µs in comparison to the simulation. In Figure 6c the simulated signal is plotted besides the deconvolved measurement of the ink-on-glass sample. For good comparability, the first peak in both curves was set as zero in time. Instead of the single broad peak of the unprocessed signal, the deconvolved data curve features a sequence of sharp peaks. The sequence of peaks, starting at 0 µs, represents the OA signal of the black ink which is subsequently followed by a series of reflections from the inner boundaries of the ink. Shortly before 0.5 µs, the reflection from the detector film back through the fronting layer to the sample and back to the detector again is visible. Approximately 1 µs later, the reflection of the glass–air boundary reaches the detector, again followed by the reflections inside the fronting layer. Finally, in the plot, the second reflection from the glass–air boundary which is created by the part of the first reflection that is reflected off the glass–ink boundary is visible. The reflection of the backing layer to air surface arrives at 3.61 µs and, thus, is not included in the plot.

The simulation is added to the graph to visualize that every feature in the measurement is accounted for in the model of the sample. For simplicity, the density of all layers was set to $1\frac{kg}{dm^{3}}$. Due to the generic nature of the sample, we refrained from implementing a multivariable optimization to find the acoustic parameters which perfectly recreate the measurement. The actual speed of sound and thickness of the black layer of ink are not very essential. Instead, it is noteworthy that this setup is low cost and the post-processing is fast, which means it can be done in real time.

In Table 1 the sound velocities and thicknesses of the different layers are listed. The speed of sound for any polymer strongly varies depending on its specific production and has to be determined for each individual piece.

## 5. Summary and Outlook

In this work, we present a simple setup using a single transparent OA detector to record acoustic nearfield signals. Signal deformation and broadening caused by limitations of the experimental setup were removed by deconvolving with the measured IRF. Due to the nearfield conditions, the IRF is directly obtained by measuring on a piece of strongly absorbing plastic and no expensive or complex sample was needed. To validate our approach, measurements on a thin layer of ink were performed and compared to simulated OA data.

While the OA raw data showed a single broad peak, the data processed with the IRF revealed individual reflections inside the ink layer. Comparison to numerical simulation showed good agreement. The relevant features of the original pressure signal are fully reproduced. The handheld setup presented in this work was built to be used for thickness determination of melanoma in the long term [11]. It is very light and can be moved in any way within the bending radius of the optical fiber used for the illumination. Thus, it fulfills the requirements on flexibility needed for in vivo measurements in dermatological clinics, for example. In the envisioned use, it is paramount for the physician to reliably see where the measurements take place. Also, even though the deconvolution procedure was developed and validated for the simple 1D case, the IRF can also be used for deconvolution of OA data of more complex samples in real life application such as in vivo human skin measurements as the acousto-electrical transfer function represented by the IRF is independent of the 3D localization and nature of acoustic sources leading to the OA signal. If the approach was to be validated in the clinical environment, OA measurements on in vivo skin would have to be validated by taking skin biopsies of the measured spot and analyzing them for their structure and optical properties. This reference data would be compared to 3D OA simulations, describing the experimental and sample conditions as closely as possible. Suitable numerical approaches and models are available [10,14,15] for this task, but the availability of skin biopsies is strongly limited and their reliable and thorough optical analysis is challenging.

A frequency cut-off at 20 MHz had to be implemented to prevent background noise from dominating the signal. Especially after the deconvolution, the higher frequencies distorted the OA signal significantly, rendering it unusable. The frequency cut-off limits the axial resolution to the depth corresponding to a runtime of approximately 50 ns. This is probably caused by the relatively long ITO electrodes which can lead to high resistance and/or by the noise pickup of the detector system due to insufficient electrical shielding. By improving the geometry and conductivity of the leads and the electromagnetic shielding as well as the grounding of detector and sample, the cutoff frequency may be raised in the next steps, thus, increasing the resolution.

This setup was used in an initial clinical pilot study to determine the thickness of melanocytic nevi [16]. While the concept is promising, the main challenge appears to be the proper grounding of the patients. The measurement on a plastic sample presented here is advantageous with regard to reproducibility and limitations on the grounding of the sample. With further research towards the prevention of parasitic noise, the resolution is expected to be improved by approximately an order of magnitude in the future. Further developments can be conducted to implement a scanning system to achieve lateral spatial resolution of the samples under study, which is particularly important for biomedical studies. 

## Figures and Tables

**Table 1:** Material layers as used in the measurements and simulation. The data sheet of the Norland Optical Adhesive does not include the sound velocity. The adhesive layer thickness is estimated from empirical values obtained in applications in other fields.

Layer	Material	Sound Velocity [m/s]	Thickness [mm]
1	PMMA	2777	5.00
2	Adhesive	2000 ^1^	0.02 ^1^
3	PVDF	2250 ^2^	0.01
4	Adhesive	2000	0.02 ^1^
5	PMMA	2777	0.5
6	Ink	1500 ^1^	0.04 ^1^
7	Glass	5640	4.00

^1^ Estimated. ^2^ Datasheet Precision Acoustics
